# Novel Polyhedral Silsesquioxanes [POSS(OH)_32_] as Anthracycline Nanocarriers—Potential Anticancer Prodrugs

**DOI:** 10.3390/molecules26010047

**Published:** 2020-12-24

**Authors:** Kinga Piorecka, Jan Kurjata, Wlodzimierz A. Stanczyk

**Affiliations:** Centre of Molecular and Macromolecular Studies, Polish Academy of Sciences, Sienkiewicza 112, 90-363 Lodz, Poland; jkurjata@cbmm.lodz.pl (J.K.); was@cbmm.lodz.pl (W.A.S.)

**Keywords:** anticancer conjugates, polyhedral oligomeric silsesquioxanes, anthracyclines

## Abstract

Anthracyclines belong to the anticancer drugs that are widely used in chemotherapy. However, due to their systemic toxicity they also exert dangerous side effects associated mainly with cardiovascular risks. The pathway that is currently often developed is their chemical and physical modification via formation of conjugated or complexed prodrug systems with a variety of nanocarriers that can selectively release the active species in cancer cells. In this study, six new nanoconjugates were synthesized with the use of polyhedral oligosilsesquioxanes [POSS(OH)_32_] as nanocarriers of the anticancer drugs anthracyclines—doxorubicin (DOX) and daunorubicin (DAU). These prodrug conjugates are also equipped with poly(ethylene glycol) (PEG) moieties of different structure and molecular weight. Water-soluble POSS, succinic anhydride modified (SAMDOX and SAMDAU) with carboxylic function, and PEGs (PEG1, PEG2 and PEGB3) were used for the synthesis. New nanoconjugates were formed via ester bonds and their structure was confirmed by NMR spectroscopy (^1^H-NMR, ^13^C-NMR, ^1^H-^13^C HSQC, DOSY and ^1^H-^1^H COSY), FTIR and DLS. Drug release rate was evaluated using UV-Vis spectroscopy at pH of 5.5. Release profiles of anthracyclines from conjugates **4**–**9** point to a range of 10 to 75% (after 42 h). Additionally, model NMR tests as well as diffusion ordered spectroscopy (DOSY) confirmed formation of the relevant prodrugs. The POSS-anthracycline conjugates exhibited prolonged active drug release time that can lead to the possibility of lowering administered doses and thus giving them high potential in chemotherapy. Drug release from conjugate **7** after 42 h was approx. 10%, 33% for conjugate **4**, 47% for conjugate **5**, **6**, **8** and 75% for conjugate **9**.

## 1. Introduction

We are currently witnessing a rapid development of anticancer drug delivery systems [1,2], exploiting such nanocarriers as, e.g., gold and mesoporous silica nanoparticles, polymers, and dendrimers [3]. Nanotechnology and nanomedicine [4,5] now offer a variety of systems that are intensively studied as delivery vehicles, modulating cytotoxicity and mediating sustained drug release in tumour tissues.

Anthracycline chemotherapy with antibiotics such as doxorubicin (DOX) and daunorubicin (DAU) plays an important role in treating many types of cancer. They exhibit wide action spectrum, high efficacy and are used in the form of soluble hydrochlorides.

**Figure 1:**
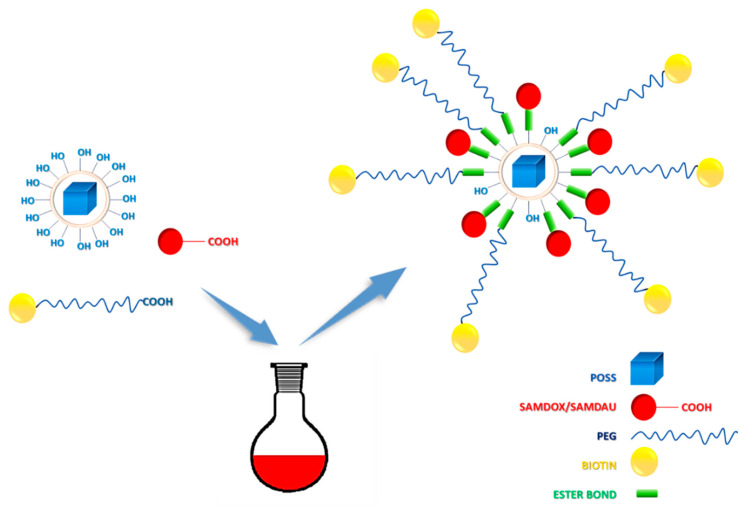
Synthetic scheme of [POSS(OH)_32_] conjugates with anthracyclines, PEG and biotin.

**Figure 2:**
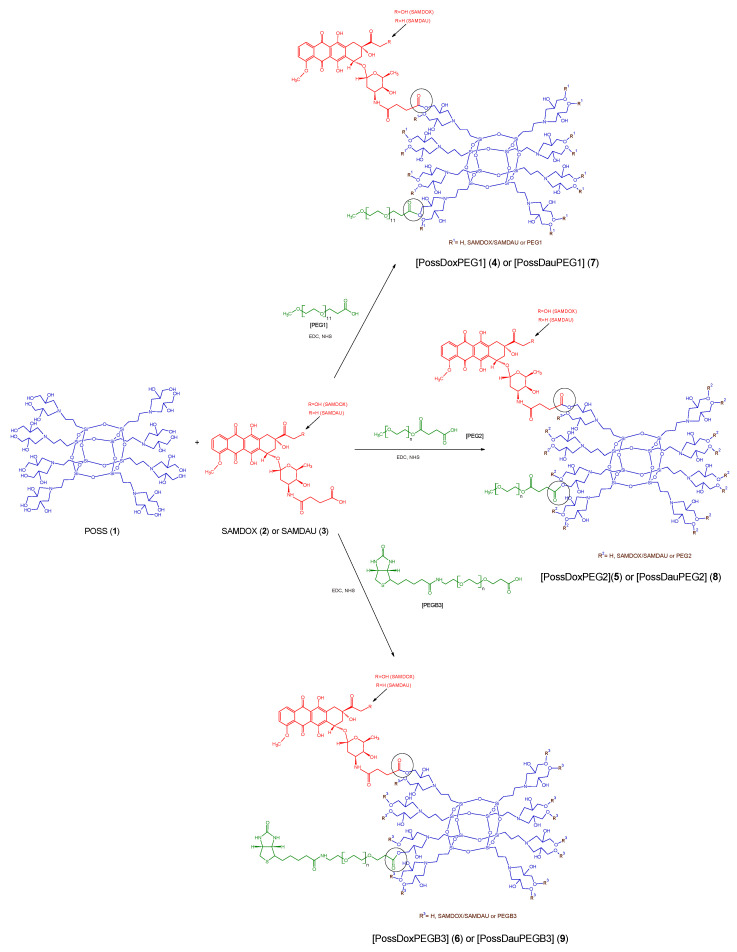
Synthesis of POSS conjugates **4**–**9**. PossDoxPEG1 (**4**): POSS conjugate containing doxorubicin and PEG1 [*O*-(2-Carboxyethyl)-*O*′-methyl-undecaethylene glycol; M_w_ 588.68), PossDoxPEG2 (**5**): POSS conjugate containing doxorubicin and PEG2 [*O*-Methyl-*O*′-succinylpolyethylene glycol 2′000; M_w_ ~2100], PossDoxPEGB3 (**6**): POSS conjugate containing doxorubicin and PEGB3 [*O*-[2-(Biotinyl-amino)ethyl]-*O*′-(2-carboxyethyl)polyethylene glycol; M_w_ 3000], PossDauPEG1 (**7**): POSS conjugate containing daunorubicin and PEG1, PossDauPEG2 (**8**): POSS conjugate containing daunorubicin and PEG2, PossDauPEGB3 (**9**): POSS conjugate containing daunorubicin and PEGB3.

They intercalate with DNA, inhibiting proliferation of cancer cells and leading to their apoptosis. Unfortunately, they are not selective and administration of high doses can often lead to heart failure [6]. Nanotechnology is one of the ways to increase the selectivity of chemotherapy via limiting systemic toxicity. The use of nanoparticles has been shown to improve the selective transport of drugs and their accumulation in the tumour, which in turn can lead to dose reduction [7]. Encapsulation, complexation or conjugation of these drugs with nanocarriers leads to slow release of the active drug, extending its time of action, leading to decreased doses. Our previous studies have been devoted to the use of polyhedral oligosilsesquioxanes as nanocarriers of anti-cancer drugs [8,9,10,11]. Silsesquioxanes are well known to facilitate cell membrane penetration [12], an important feature in drug delivery. Additionally, they are biocompatible [13] and nontoxic [14]. The current research presents results on application of hydrophilic silsesquioxanes, known in the literature as [POSS(OH)_32_], as novel anthracycline nanocarriers, synthesized by hydrolytic condensation of a functionalized precursor—*N,N*-di(2,3-dihydroxypropyl)(aminopropyl)triethoxy-silane [15]. Contrary to the previously used POSSs, the silsesquioxanes obtained from *N*,*N*-di(2,3-dihydroxypropyl)(aminopropyl)triethoxysilane have relatively narrow size distribution and a large number of functional OH moieties. The average particle diameter of these water soluble structures is on average ~3.0 nm and the synthesis of such a system is simple and effective. Additionally, due to partial coupling of hydroxyl groups of the carrier with carboxy terminated, methoxylated poly(ethylene glycol) (PEG), apart from esterification with succinic anhydride modified anthracyclines [16], the solubility in aqueous media can be largely improved. It also allows additional binding of the targeting moiety biotin (Figure 1 and Figure 2).

In this work, 6 POSS-anthracycline conjugates were synthesized: POSS conjugate containing doxorubicin and PEG1 [*O*-(2-carboxyethyl)-*O*′-methyl-undecaethylene glycol; M_w_ 588.68]—PossDoxPEG1 (**4**), doxorubicin and PEG2 [*O*-Methyl-*O*′-succinylpolyethylene glycol 2′000; M_w_ ~2100]—PossDoxPEG2 (**5**), doxorubicin and PEGB3 [*O*-[2-(Biotinyl-amino)ethyl]-*O*′-(2-carboxyethyl)polyethylene glycol; M_w_ 3000]—PossDoxPEGB3 (**6**), daunorubicin and PEG1—PossDauPEG1 (**7**), daunorubicin and PEG2—PossDauPEG2 (**8**) and daunorubicin and PEGB3—PossDauPEGB3 (**9**) (Figure 2). The research was aimed to develop effective synthetic methods of silsesquioxane nanoconjugates with anticancer drugs. Such conjugates would be capable of releasing active drugs in cancer cells. The structure of all synthesized nanoconjugates was confirmed by NMR spectroscopy, FTIR, and DLS.

## 2. Results and Discussion

### 2.1. Model Reaction

The model reaction (Figures S2 and S3 and Table S1) was carried out to allow interpretation of complex NMR spectra of nanoconjugates (**4**–**9**). The POSS structure—T^8^[(CH_2_)_2_S(CH_2_)_2_OH]_8_ applied in the model reaction has a well-defined structure, in contrast to [POSS(OH)_32_] being a mixture of cage silsesquioxanes. It was obtained by modifying commercial POSS-Vi with 2-mercapotetanol. In addition, PEG1 with a lower molecular weight was used, which simplifies interpretation of the NMR spectra. The post reaction mixture was pre-purified (see Section 3), and its composition was determined using ^1^H-^1^H COSY and ^1^H-^13^C HSQC NMR spectra of the substrates and the product. Thus, in the subsequent syntheses, it was possible to determine whether the purified products also contained free drugs and/or free PEGs.

### 2.2. Determination of Total Drug Content in Nanoconjugates **4**–**9**

**Figure 3:**
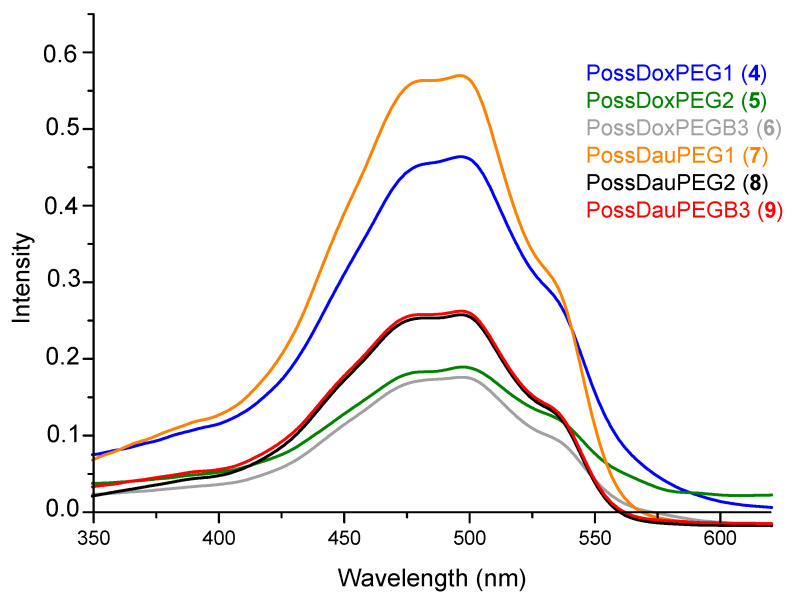
Measurement of maximum contents of DOX and DAU released from the relevant conjugates (**4**–**9**) (by mixing with HCl (36%) at 50 °C).

Total drug content was measured using UV-Vis spectroscopy (Figure 3). Nanoconjugates (**4**–**9**) (1 mg) were dissolved in H_2_O/DMF (1 mL, 5:1), then 200 µL of this solution was taken and diluted with 3 mL of solvent (H_2_O/DMF (5:1)), followed by mixing with 0.2 mL HCl (36%). It was kept at 50 °C for 2 h and then at room temperature for 24 h. A calibration curve for DOX/DAU was constructed and total drug content was calculated from UV-Vis spectra of DOX, DAU and all the nanoconjugates (**4**–**9**) at 480 nm (Figures S4 and S5).

Table 1 shows that conjugate **4** (42.88%) has the highest anthracycline content and conjugate **6** (16.38%) the lowest. Conjugate **4** (88.0%) exhibits the highest efficiency of drug attachment, while conjugate **5** is characterized by the lowest (47.4%). The PEG1-containing conjugates (**4** and **7**) are most effective in conjugation with anthracyclines. In contrast, the conjugates containing PEGB3 in the molecule show the lowest efficiency of binding drugs. This may be related to the molecular mass and structure of PEGs—PEGB3 has the highest molecular mass, thus slowing down the ester bond formation.

### 2.3. Drugs Release Study

**Figure 4:**
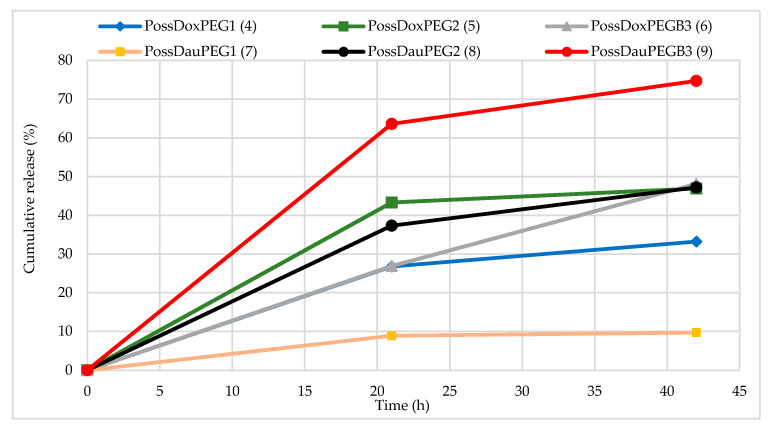
In vitro DOX/DAU release profiles from nanoconjugates in citrate buffer solution at 310 K quantified by UV-Vis method.

DOX/DAU release from the nanoconjugates was determined in citrate buffer solution—(pH 5.5 0.1 M) at 310 K. Nanoconjugates, dissolved in DMF, were placed in a dialysis bag (MWCO: 2 kD, Spectrum Laboratories) and inserted into buffer solution (conjugate concentrations are described in the Table S3). The amount of released drug was calculated from the UV-Vis spectra. The drug release profile of PossDauPEGB3 (**9**) conjugate showed the fastest release of DAU in an acid environment. In this case, over 60% of POSS conjugated DAU was released within 21 h and over 70% within 42 h. Release profiles of anthracyclines from conjugates **4**–**6** and **8** point to a maximum amount after 42 h in the range of 33 to 47%. Conjugate **7** showed the lowest drug release profile (only ~10% after 21 h and 42 h). As can be seen in Figure 4, the drug release profiles from the conjugates with PEG1 indicate the lowest release values, and are not dependent only on the type of applied PEG. It can be also related to the complexation of drugs and PEGs (not covalently bound to POSS). Conjugate **9** has a biotynyl fragment that can form drug complexes with NH moieties via hydrogen bonds. Non-covalent systems involving anthracyclines are known to be formed and are cleaved easier than relevant conjugates [1]. The hydrogen bonded anthracyclines and POSS(OH)_32_ complexes have been recently described [17]. Additionally, Figure S6 shows the graphs of absorbance intensity measured with UV-Vis for drug release from the relevant conjugates after 21 h and 42 h. It is evident that the drug release rate from conjugates is slow enough to make them potentially good candidates for anti-cancer therapy [17].

As the plots in Figure 4 prove, the conjugates (**4** and **7**) containing PEG1 in their structure show the slowest rate release of the drugs, while the PEG2-containing conjugates show higher and similar release profiles. This may originate from the fact that drug content in **4** and **7** is the highest amongst all the synthesized systems.

### 2.4. Nuclear Magnetic Resonance (NMR)—^1^H-^13^C HSQC

**Figure 5:**
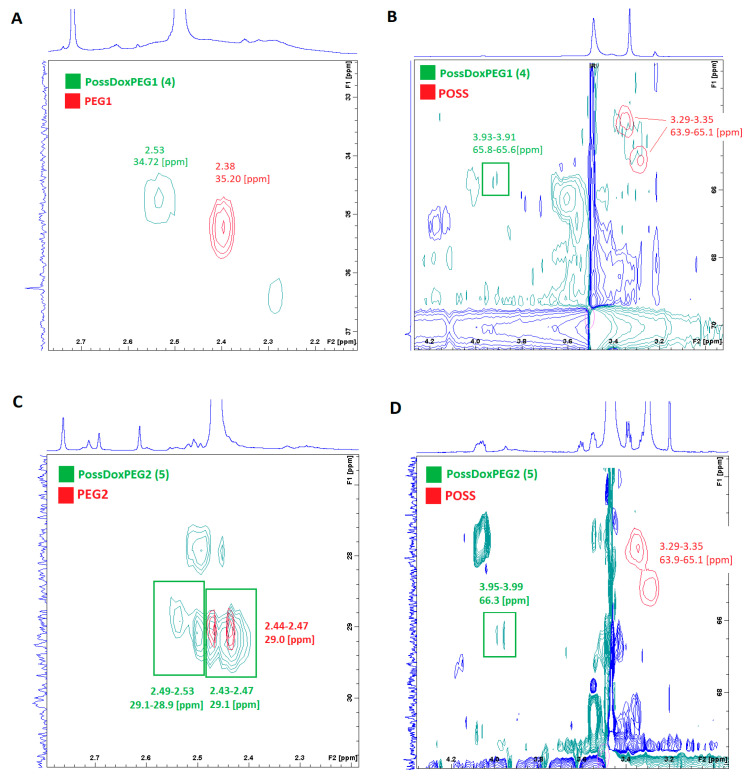

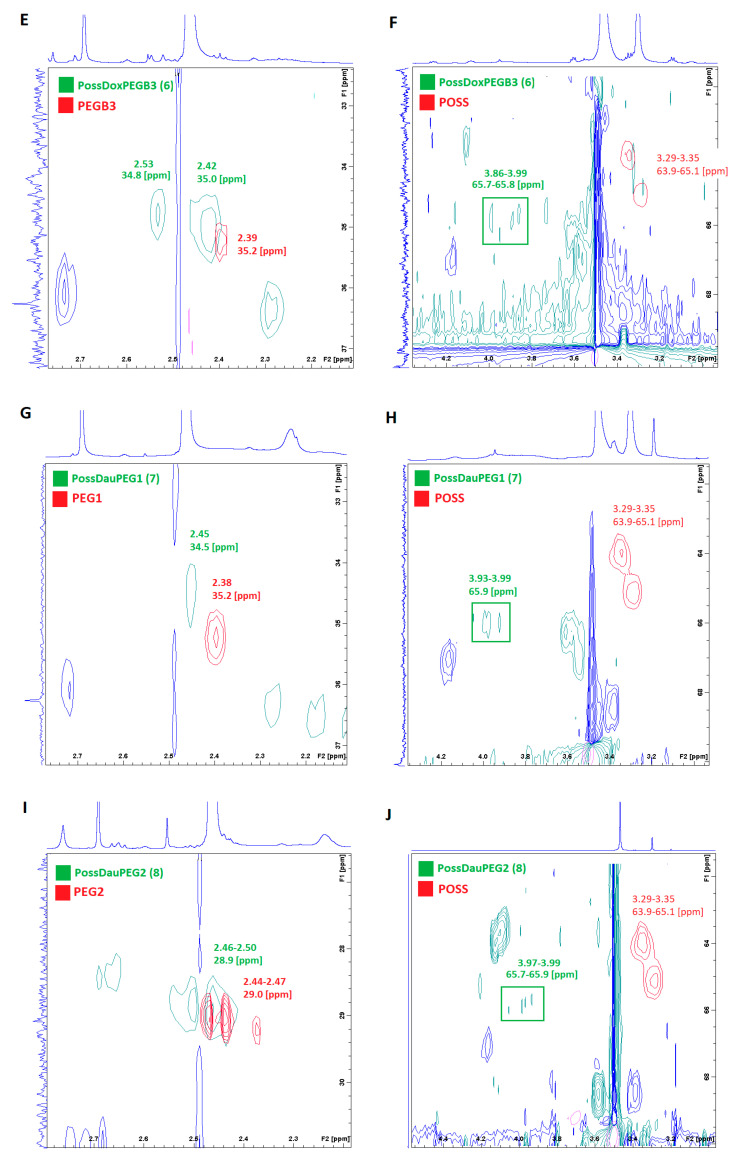

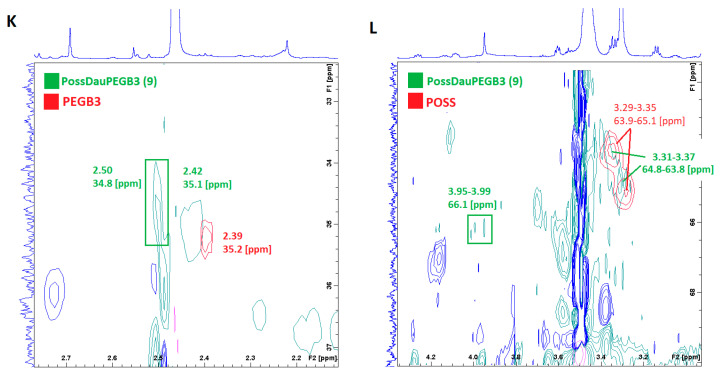
Superimposition of the ^1^H-^13^C HSQC spectra of the substrates (PEG and POSS) on the ^1^H-^13^C HSQC spectra of the products (conjugates **4**–**9**). (**A**,**B**) PossDoxPEG1 (**4**); (**C**,**D**) PossDoxPEG2 (**5**); (**E**,**F**) PossDoxPEGB3 (**6**); (**G**,**H**) PossDauPEG1 (**7**); (**I**,**J**) PossDauPEG2 (**8**); (**K**,**L**) PossDauPEGB3 (**9**).

Basing on the ^1^H-^13^C HSQC spectra, we were able to confirm the formation of ester bonded conjugates. Figure 5 shows the ^1^H-^13^C HSQC spectra of the conjugates superimposed on the spectra of the substrates (PEG and POSS). It points to differences in proton shift values associated with ester bond formation. The PEG1 protons chemical shift (CH_2_COOH) moved towards the lower field (from 2.38 to 2.53 ppm for conjugate **4** and from 2.38 to 2.45 ppm for conjugate **7**) on formation of the ester bonds. The PEG2 protons chemical shift (CH_2_COOH) also changed towards the lower field from ~2.4 to ~2.5 ppm for conjugates **5** and **8**. The same also applies to PEGB3 (a shift from 2.39 to 2.53 ppm for conjugate **6** and from 2.39 to 2.50 ppm for conjugate **9**). In order to investigate differences in chemical shifts of POSS before and after ester bond formation, POSS spectra were superimposed on the conjugates spectra. Based on the spectra, it can be concluded that in conjugates **4**, **6** and **9**, there is incomplete substitution of the hydroxyl groups. This is evidenced by additional cross peaks at the chemical shift values overlapping with the POSS (-CH_2_OH) proton shift values −3.29–3.35 ppm.

**Figure 6:**
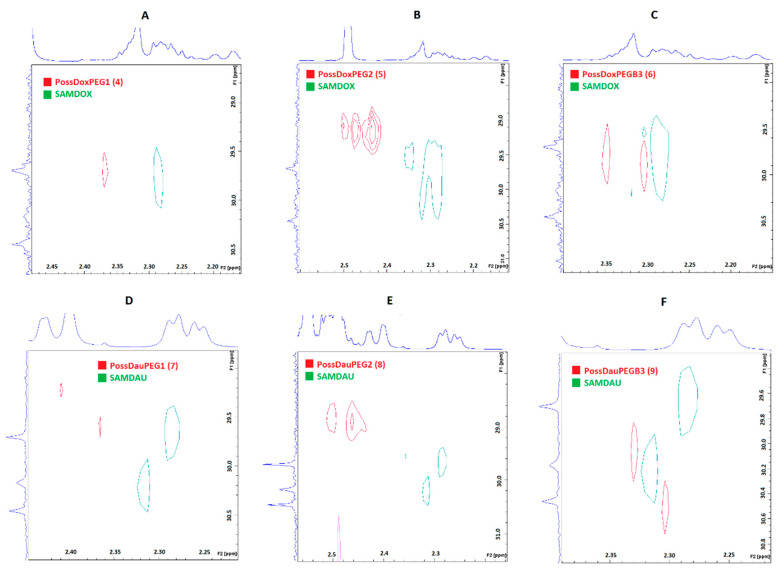
Superimposition of the ^1^H-^13^C HSQC spectra of the substrates (SAMDOX/SAMDAU) on the ^1^H-^13^C HSQC spectra of the products (conjugates **4**–**9**). (**A**) PossDoxPEG1 (**4**); (**B**) PossDoxPEG2 (**5**); (**C**) PossDoxPEGB3 (**6**); (**D**) PossDauPEG1 (**7**); (**E**) PossDauPEG2 (**8**); (**F**) PossDauPEGB3 (**9**).

Figure 6 shows the superimposed ^1^H-^13^C HSQC spectra of the conjugates on the SAMDOX and SAMDAU spectra to investigate differences in chemical shifts of the anthracycline protons adjacent to the ester/carboxylic functions after (red) and before (green—SAMDOX and SAMDAU) the conjugation. The formation of an ester bond is associated with a change in the chemical shifts of adjacent protons (POSS-antracycline-CH_2_CH_2_-ester bond) towards the lower field. For conjugates 5 and 8 containing PEG2 in their molecule, this is a marked change from ~2.3 ppm to ~2.5 ppm. In contrast to the ^1^H-^13^C HSQC spectra shown after superimposing PEG/POSS on the spectra of the conjugates (Figure 5), the ^1^H-^13^C HSQC spectra (Figure 6) did not give an unequivocal answer as to whether SAMDOX and SAMDAU are incorporated into the product.

### 2.5. Diffusion NMR Spectroscopy

**Figure 7:**
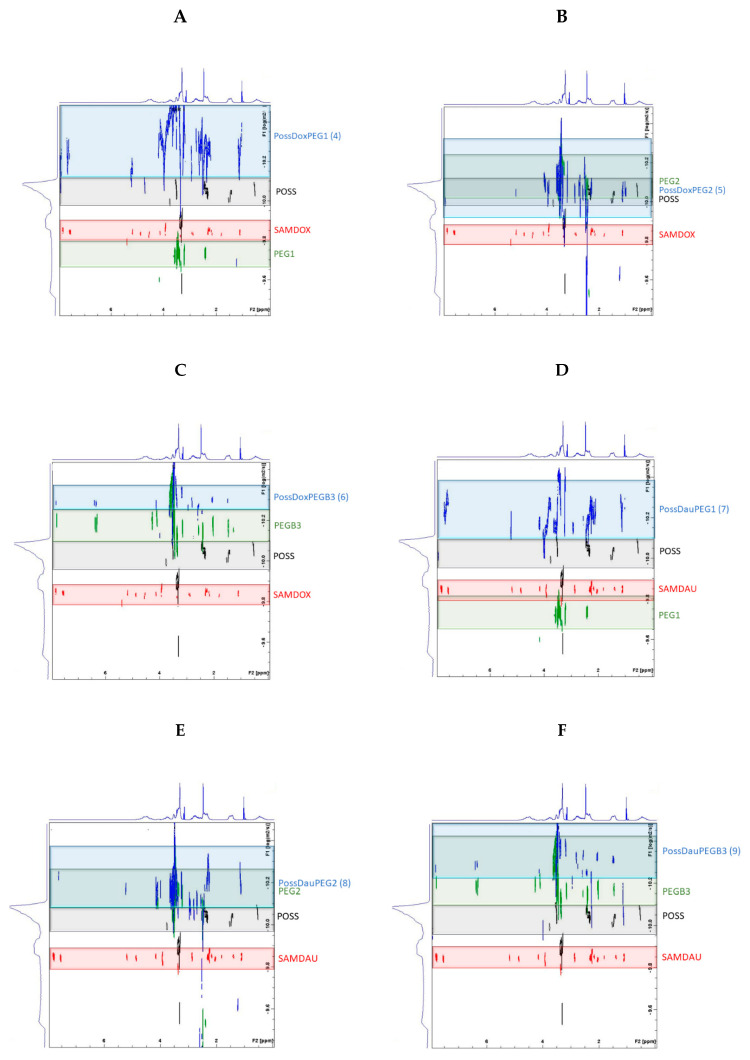
Superimposition of the DOSY spectra of the substrates (SAMDOX/SAMDAU, PEG1/PEG2/PEGB3 and POSS(OH)_32_) on the DOSY spectra of the products (conjugates **4**–**9**). (**A**) PossDoxPEG1 (**4**); (**B**) PossDoxPEG2 (**5**); (**C**) PossDoxPEGB3 (**6**); (**D**) PossDauPEG1 (**7**); (**E**) PossDauPEG2 (**8**); (**F**) PossDauPEGB3 (**9**).

**Figure 8:**
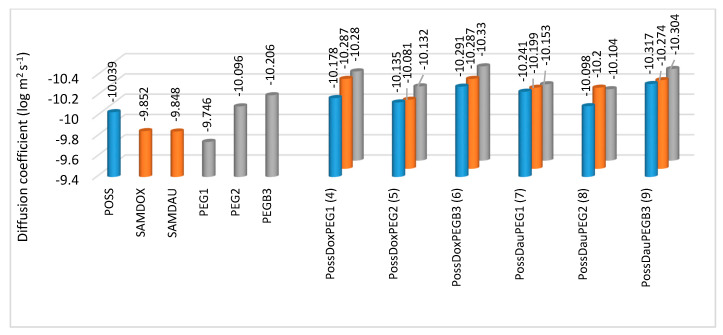
Self-diffusion coefficients (log m^2^s^−1^) of the SAMDOX, SAMDAU, POSS(OH)_32_, PEG1, PEG2, PEGB3 and conjugates **4**–**9** from the resonance signals: POSS (1.45 ppm), SAMDOX (7.84 ppm), SAMDAU (7.79 ppm), PEG1 (2.43 ppm), PEG2 (3.49 ppm), PEGB3 (3.48 ppm), PossDoxPEG1 (1.42 ppm; 7.85 ppm; 2.43 ppm), PossDoxPEG2 (1.42 ppm; 7.83 ppm; 3.49 ppm), PossDoxPEGB3 (1.47 ppm; 7.84 ppm; 3.43 ppm), PossDauPEG1 (1.45 ppm; 7.79 ppm; 2.42 ppm), PossDauPEG2 (1.49 ppm; 7.78 ppm; 3.52 ppm) and PossDauPEGB3 (1.49 ppm; 7.82 ppm; 3.51 ppm).

Diffusion NMR spectroscopy (DOSY) was performed to further confirm the formation of the conjugates **4**–**9**. The results were determined by superimposing the DOSY spectra of the substrates (SAMDOX/SAMDAU, PEG1/PEG2/PEGB3 and POSS(OH)_32_) on the DOSY spectrum of the products (conjugates **4**–**9**) (Figure 7). Self-diffusion coefficients (D) of the SAMDOX, SAMDAU, POSS(OH)_32_, PEG1, PEG2, PEGB3 and conjugates **4**–**9** were determined from the resonance signals: POSS (1.45 ppm), SAMDOX (7.84 ppm), SAMDAU (7.79 ppm), PEG1 (2.43 ppm), PEG2 (3.49 ppm), PEGB3 (3.48 ppm), PossDoxPEG1 (1.42 ppm; 7.85 ppm; 2.43 ppm), PossDoxPEG2 (1.42 ppm; 7.83 ppm; 3.49 ppm), PossDoxPEGB3 (1.47 ppm; 7.84 ppm; 3.43 ppm), PossDauPEG1 (1.45 ppm; 7.79 ppm; 2.42 ppm), PossDauPEG2 (1.49 ppm; 7.78 ppm; 3.52 ppm) and PossDauPEGB3 (1.49 ppm; 7.82 ppm; 3.51 ppm). The results are presented in Figure 8. Differences in diffusion rates of substrates and conjugates (**4**–**9**) point to the formation of larger conjugated molecules, as they migrate at a smaller rate than the relevant substrates, due to a lower value of self-diffusion coefficients.

### 2.6. Fourier Transform Infrared Spectroscopy

**Figure 9:**
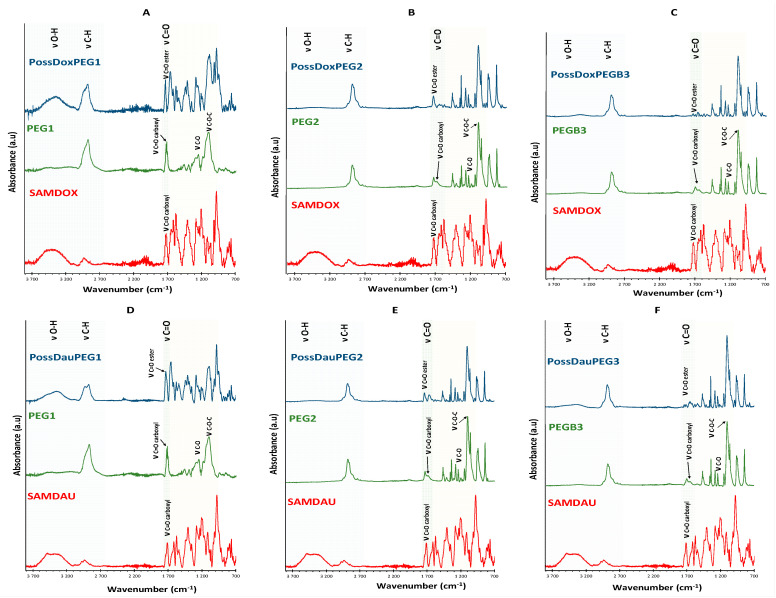
Fourier transform infrared spectra of conjugates **4**–**9** compared with those of antracyclines and PEGs. Blue color—range 2700–3700 cm^−1^ (ν_O-H_, ν_C-H_); green color—range 1700–1750 cm^−1^ (ν_C=O_, ester). (**A**) PossDoxPEG1 (**4**); (**B**) PossDoxPEG2 (**5**); (**C**) PossDoxPEGB3 (**6**); (**D**) PossDauPEG1 (**7**); (**E**) PossDauPEG2 (**8**); (**F**) PossDauPEGB3 (**9**).

FTIR spectroscopy was additionally used for structural characterization of the conjugates formed via ester bond between drugs and the POSS carrier. FTIR spectra of conjugates differed from that of SAMDOX/SAMDAU [11]. The spectra of conjugates **4**–**9** (Figure 9) show a new absorption frequency at ~1734 cm^−1^ (ν_C=O_, ester bond) indicating formation of an ester bond between SAMDOX/SAMDAU and the POSS hydroxyl groups.

**Figure 10:**
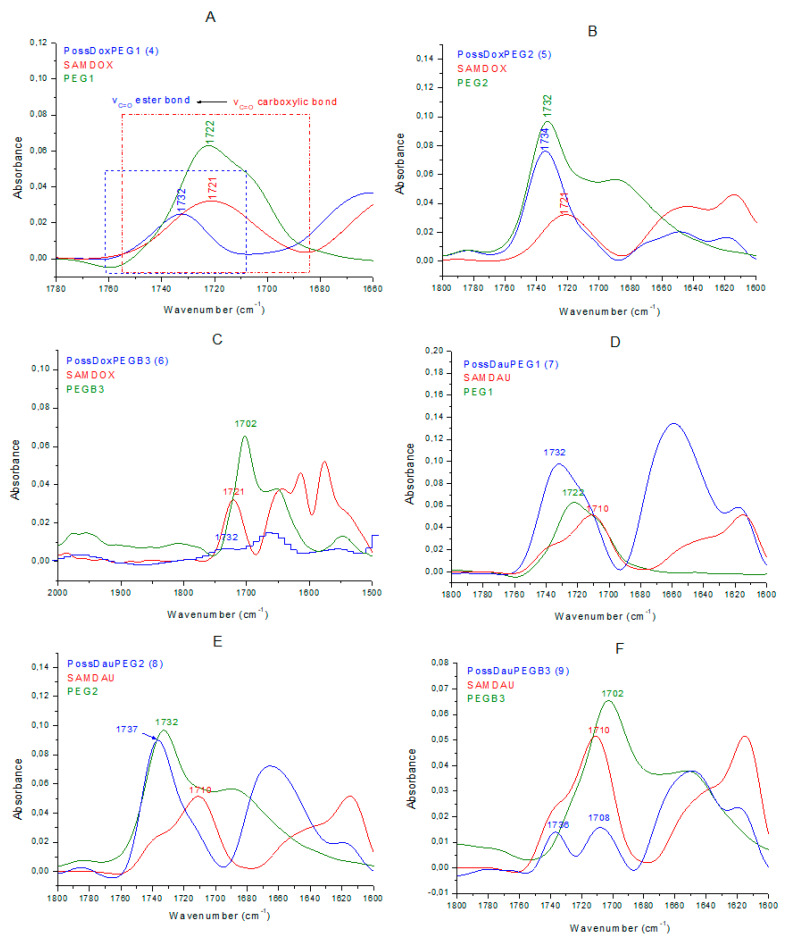
FTIR spectra of conjugates **4**–**9** compared with the ones of anthracyclines and PEGs (vibration range—ν_c=o,_ ester bond). (**A**) PossDoxPEG1 (**4**); (**B**) PossDoxPEG2 (**5**); (**C**) PossDoxPEGB3 (**6**); (**D**) PossDauPEG1 (**7**); (**E**) PossDauPEG2 (**8**); (**F**) PossDauPEGB3 (**9**)

Conjugate **4** (Figure 10A) exhibits the new absorption band at 1732 cm^−1^ (ν_C=O_, ester bond), clearly indicating the formation of ester bond between SAMDOX/PEG1 and POSS(OH)_32_. There is no absorption in the spectrum indicating the presence of unreacted carboxyl groups (SAMDOX 1721 cm^−1^ (ν_C=O_ carboxylic bond) and PEG1 1722 cm^−1^ (ν_C=O_ carboxylic bond)). The same characteristic absorption is also evident for conjugate **6** (Figure 10C).

The spectrum of conjugate **5** (Figure 10B) is more difficult to interpret as PEG2 also has an ester linkage. We can assume that the ester bond in conjugate **5** has been formed, as shown by the shift of the ν_C=O_ PEG2 (ester bond) absorption from 1732 cm^−1^ to 1734 cm^−1^. There is no absorption in the spectrum that would indicate the presence of unreacted carboxyl groups. The same conclusion can be drawn from the spectrum of conjugate **8** (Figure 10E).

In contrast, in the spectrum of conjugate **7** (Figure 10D), the absorption at 1732 cm^−1^ (ν_C=O_ ester bond), indicating the formation of ester bond, also appears, but there are still unreacted carboxyl groups from SAMDAU or PEG1 as shown by widening of the band at 1732 cm^−1^. The situation is similar for conjugate **9** (Figure 10F), however, here there is a clear absorption at 1708 cm^−1^ indicating unreacted –COOH moieties from SAMDAU or PEGB3.

### 2.7. Hydrodynamic Diameters of Conjugates **4**–**9**

**Figure 11:**
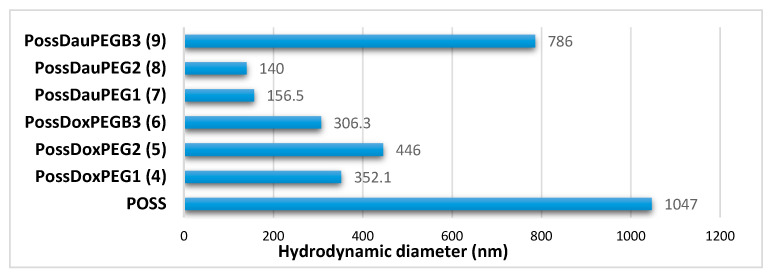
Comparison of hydrodynamic diameters of POSS(OH)_32_ and conjugates **4**–**9**.

The hydrodynamic diameter of the POSS(OH)_32_ and conjugates **4**–**9** was investigated by DLS. Figure 11 shows that POSS has the highest hydrodynamic diameter, which may suggest that its hydroxyl groups form a network of hydrogen bonds. The involvement of the POSS hydroxyl groups in the formation of ester bonds in conjugates **4**–**9** resulted in a reduction in their hydrodynamic diameter.

## 3. Materials and Methods

### 3.1. Materials

Doxorubicin (DOX) and daunorubicin (DAU) (Beijing Packbuy M&C, Beijing, China), succinic anhydride (Sigma-Aldrich, Saint Louis, MO, USA), *N*-hydroxysuccinimide (NHS) (Sigma-Aldrich), 1-ethyl-3-(3-dimethylaminopropyl) carbodiimide hydrochloride (EDC) (Sigma-Aldrich), PEG1: *O*-(2-Carboxyethyl)-*O*′-methyl-undecaethylene glycol [Molecular Weight 588.68; *n* = 11], PEG2: *O*-Methyl-*O*′-succinylpolyethylene glycol 2′000 [M_r_ ~2100], PEGB3: *O*-[2-(Biotinyl-amino)ethyl]-*O*′-(2-carboxyethyl)polyethylene glycol [M_p_ 3000] (Sigma-Aldrich) were used as supplied. Triethylamine (Et_3_N, CHEMPUR, Piekary Slaskie, Poland), methylene chloride (POCh) and *N,N*-dimethylformamide (DMF, POCh, Gliwice, Poland) were purified as described in the literature [18]. Hydroxyl functionalized silsesquioxane cage POSS(OH)_32_ was synthesized according to the method described previously in the literature [19]. The reproducibility of synthesized structures was proven by three independent experiments [17]. Syntheses of succinic anhydride-modified daunorubicin (SAMDAU) and succinic anhydride-modified doxorubicin were performed according to the methods described in the literature [16] (Figure S1).

### 3.2. General Remarks

Ultraviolet–visible (UV–Vis) measurements were carried out using the Specord S600 spectrophotometer using 10 mm path length quartz cuvettes. Solutions of conjugates at concentration C_conjugates_ = 0.05882 mg/mL were prepared in water/DMF (5:1). DOX/DAU release from the nanoconjugates was determined at a pH of 5.5 (citrate buffer solution—0.1 M) at 310 K (Table S3).

^1^H, ^13^C, ^1^H-^13^C HSQC and DOSY nuclear magnetic resonance (NMR) spectra were recorded using Bruker Avance III 500 MHz instrument (Bruker BioSpin GmbH, Rheinstetten, Germany). Chemical shifts are reported in ppm downfield from TMS using DMSO-d6 as a solvent and at 295 K (^1^H, ^13^C, ^1^H-^13^C HSQC, ^1^H-^1^H COSY) and 298 K (DOSY).

FTIR (Fourier transform infrared spectroscopy) spectra were obtained using a Nicolet 6700 spectrometer equipped with a deuterated triglycine sulfate detector and using attenuated total reflectance (ATR) for 64 scans at a 2cm^−1^ resolution.

Hydrodynamic diameters of conjugates **4**–**9** measured by dynamic light scattering (DLS) in deionized water (with 5% *v*/*v* DMF) at 298 K. DLS studies were performed using a ZetaSizer Nano ZS (Malvern Instruments, Malvern, UK) equipped with HeNe red laser (λ = 633 nm) at a measurement angle of 173°. DLS measurements were carried out at the concentration of pure POSSOH_32_ (C_POSS_ = 1.934 × 10^−3^ mg/mL) and of the conjugates **4**–**9** (C_conjugates_ = 1.187 × 10^−3^ mg/mL).

### 3.3. General Synthesis of Nanoconjugates **4**–**9**

All of the synthetic steps were carried out in the dark (Figure 2). Table S2 shows the concentrations of the reagents used in the reaction. SAMDOX/SAMDAU, NHS and EDC were placed in a flask, dissolved in DMF and stirred under nitrogen. At the same time, a PEG, NHS and EDC mixture was prepared in DMF and stirred under nitrogen. Both reaction mixtures were stirred for 3 h at room temperature. Then both mixtures were added dropwise to the POSS solution in DMF and the reaction mixture was stirred for 5 days at room temperature, filtered, concentrated under reduced pressure (rotary evaporator) and dialised in molecular porous membranę tubing (MWCO: 3.5 kD, Standard RC Tubing, Spectrum Laboratories) in DMF for 1 week (DMF was changed three times). At the end the final products were dried on a vacuum line.

### 3.4. Model Reaction

Synthesis of POSS T_8_[(CH_2_)_2_S(CH_2_)_2_OH]_8_. Octa(vinyl)silsesquioxane (T_8_-Vi) (2.00 g, 3.1593 × 10^−3^ mol) and 2-mercaptoethanol (2.5 mL), 2,2-dimethoxy-2-phenylacetophenone (DMPA) (0.1295 g) were introduced into a quartz reactor and dissolved in THF (30 mL). The reaction mixture was irradiated with a UV lamp at 350 nm for 1.5 h. Volatiles were removed under vacuum. The residue was dissolved in THF (5 mL), precipitated in pentane (10 × 30 mL), separated, washed with pentane (100 mL) and dried under vacuum.

Synthesis of POSSDAU-MR. The reaction carried out in the dark. SAMDAU, PEG1, NHS and EDC were placed in a flask, dissolved in DMF and stirred under nitrogen for 17 h at room temperature. After this time the mixture was added dropwise to the POSS solution in DMF and the reaction mixture was stirred for 3 days at room temperature. Then the reaction mixture was concentrated under reduced pressure and the product was purified on a column Sephadex LH20 (length 55 cm, ø (diameter of the column) 1 cm, eluent: dry DMF). At the end the product was dried under vacuum.

## 4. Conclusions

Six new nanoconjugates were synthesized using polyhedral oligosilsesquioxanes (POSS) as nano-carriers for anthracyclines (DOX and DAU) and PEGs (water solubilizing agent). The new conjugates contain an ester bond capable of hydrolysis under the conditions of lowered pH (5.5), characteristic for cancer cells. The structure of the new prodrugs were confirmed by NMR (^1^H-NMR, ^13^C-NMR, ^1^H-^13^C HSQC and DOSY), FTIR and DLS. The analytical methods applied in this work can serve as the important tools and model approach in studies of other nanocarrier-anthracycline conjugates. This is evidenced by the disappearance of the signal at ~2.5 ppm (CH_2_OH) associated with the formation of an ester bond (in the NMR analysis) and the appearance of the 1730 cm^−1^ peak in the FTIR analysis. Using simple and efficient NMR techniques, it was possible to detect formation of the prodrug conjugates. The conjugates are larger in size, compared to anthracycline antibiotics themselves, which is favorable due to the presumably limited normal cell penetration during chemotherapy. In addition, they show longer release time, which makes them potential candidates for biomedical applications in anticancer therapy. POSS type nanocarriers were proven again [10,11] to be useful systems in formation of nanoconjugates and nanocomplexes with anthracycline drugs. The present work shall be expanded by in vitro studies as soon as the test laboratory reopens after COVID-19 closure.

## Figures and Tables

**Table 1:** Characteristics of conjugates **4**–**9**. Total drug content refers to the weight percentage of the drug in the conjugate. The calculated maximum drug content (wt%) relates to the theoretical maximum drug content—calculated from the weight of drug taken for the reaction. The drug attachment efficiency was calculated on the basis of total drug content and calculated maximum drug content.

Type of Nanoconjugate	Total Drug Content (wt%)	Calculated Maximum Drug Contents (wt%)	Drug Attachment Efficiency (wt%)
PossDoxPEG1 (**4**)	42.88%	48.70%	88.0%
PossDoxPEG2 (**5**)	17.54%	37.02%	47.4%
PossDoxPEGB3 (**6**)	16.38%	27.00%	60.7%
PossDauPEG1 (**7**)	42.33%	54.50%	77.7%
PossDauPEG2 (**8**)	19.20%	33.93%	56.6%
PossDauPEGB3 (**9**)	19.52%	27.18%	71.8%

## Supplementary Materials

The following are available online, Figure S1: Scheme for the synthesis of SAMDOX and SAMDAU; Figure S2: Scheme for the synthesis of POSSDAU-MR.; Figure S3: Structure of POSSDAU-MR; Table S1: NMR results for POSSDAU-MR.; Table S2: Concentrations of the reagents used in conjugation reaction **4**–**9**; Table S3: Concentrations of the conjugates **4**–**9** in drugs release study; Figure S4: Calibration curves of: (A) DOX in H_2_O/DMF (5:1) (B) DAU in H_2_O/DMF; Figure S5: Dependence of anthracycline concentration (A. DOX, B. DAU) on the absorbance intensity in the UV-Vis spectrum; Figure S6: A. Study of DOX/DAU release from nanoconjugates at pH 5.5 at 310 K quantified by UV-Vis method after 21 h (A) and after 42 h (B); ^1^H NMR spectra of **4**–**9** (500 MHz, 295 K, DMSO-d6).

## Sample Availability

Samples of all compounds (POSS, SAMDAU, SAMDOX, compounds **4**–**9**) are available from the authors.
