# Supplementary material for: Novel Polyhedral Silsesquioxanes [POSS(OH)32] as Anthracycline Nanocarriers—Potential Anticancer Prodrugs
Source: Molecules. 2020 Dec 24;26(1):47. doi: 10.3390/molecules26010047 (PMC7794878; doi:10.3390/molecules26010047)
Supplement: Supplementary file 1 [file molecules-26-00047-s001.pdf]

molecules

Supporting Information

Novel Polyhedral Silsesquioxanes [POSS(OH)$_{32}$] as Anthracycline Nanocarriers—Potential Anticancer Prodrugs

Kinga Piorecka [1,*], Jan Kurjata [1] and Wlodzimierz A. Stanczyk [1,]

[1] Centre of Molecular and Macromolecular Studies, Polish Academy of Sciences, Sienkiewicza 112, 90-363 Lodz, Poland, kgradzin@cbmm.lodz.pl, jkurjata@cbmm.lodz.pl, was@cbmm.lodz.pl

* Correspondence: kgradzin@cbmm.lodz.pl

Table of Content

Fig. S1. Scheme for the synthesis of SAMDOX and SAMDAU.

Fig. S2. Scheme for the synthesis of POSSDAU-MR.

Fig. S3. Structure of POSSDAU-MR

Table S1. NMR results for POSSDAU-MR.

Table S2. Concentrations of the reagents used in conjugation reaction **4–9**.

Table S3. Concentrations of the conjugates **4–9** in drugs release study.

Fig. S4. Calibration curves of: (**A**) DOX in H_2O/DMF (5:1) (**B**) DAU in H_2O/DMF

Fig. S5. Dependence of anthracycline concentration (**A**. DOX, **B**. DAU) on the absorbance intensity in the UV-Vis spectrum.

Fig. S6. A. Study of DOX/DAU release from nanoconjugates at pH 5.5 at 310 K quantified by UV-Vis method after 21 h (**A**) and after 42 h (**B**).

Fig. S7. ^{1}H-NMR spectra of **4–9** (500MHz, 295K, DMSO-d6).

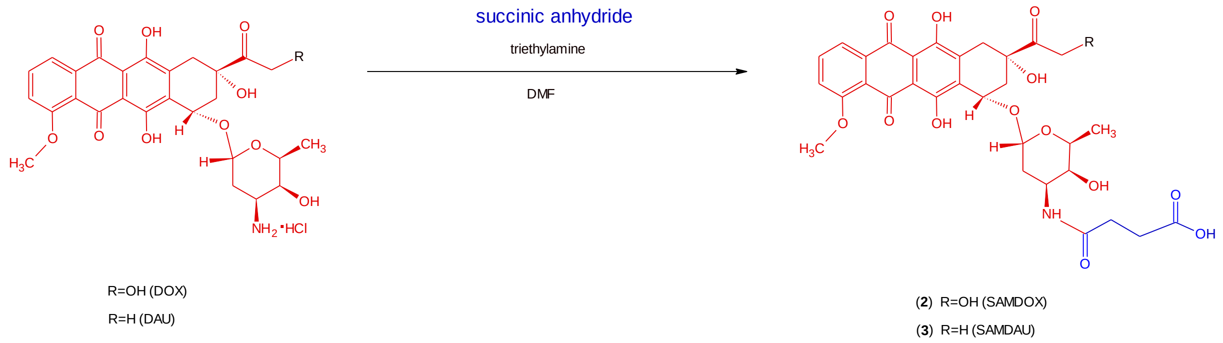

Fig. S1. Scheme for the synthesis of SAMDOX and SAMDAU.

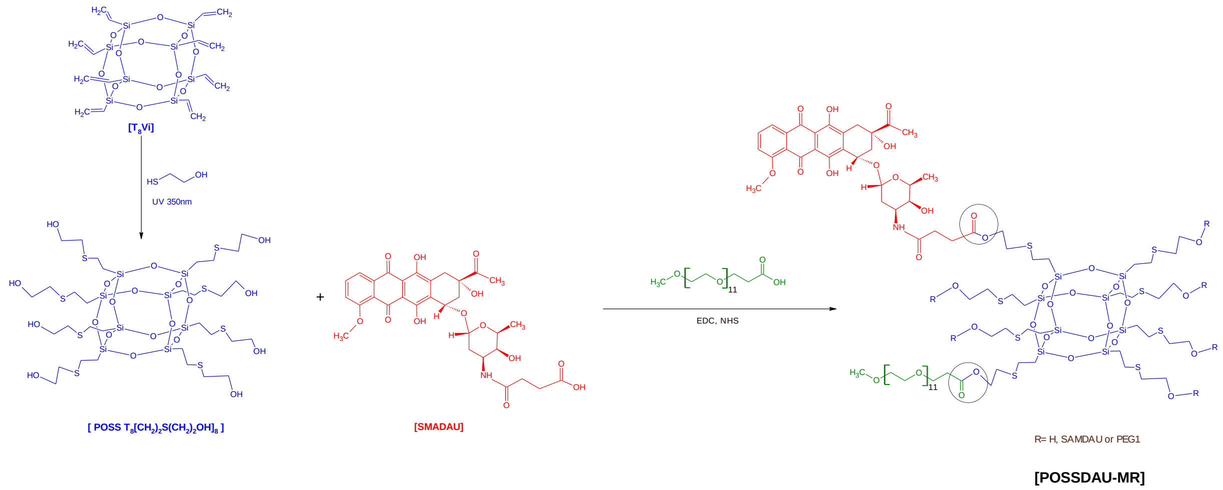

Fig. S2. Scheme for the synthesis of POSSDAU-MR.

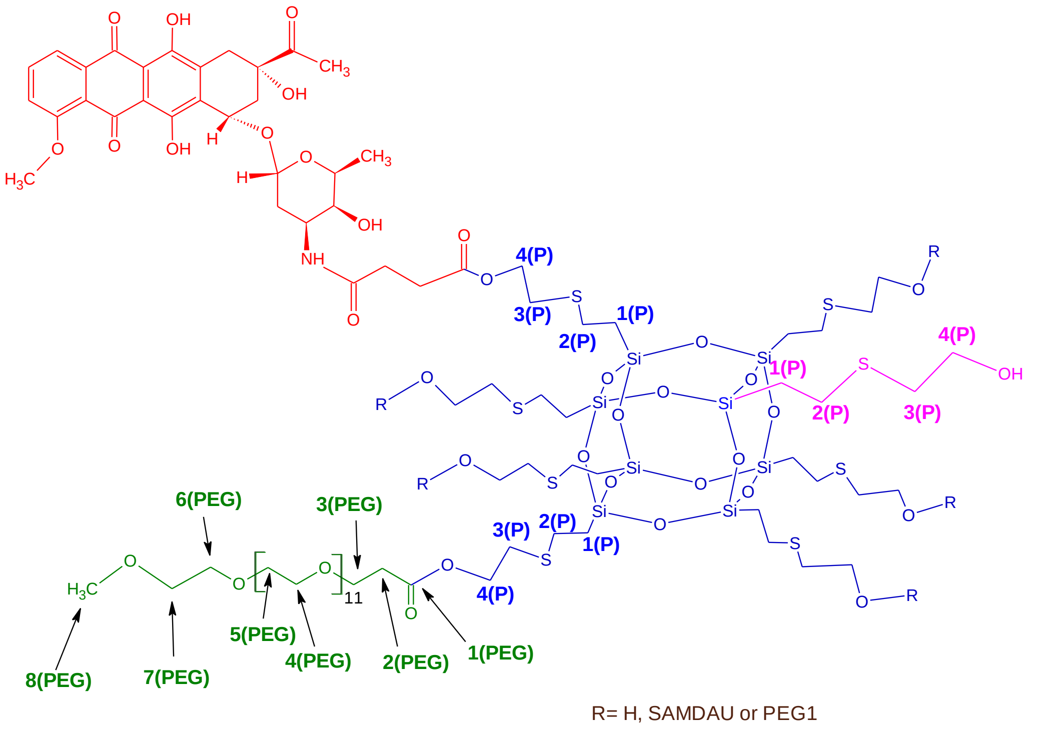

[POSSDAU-MR]

Fig. S3. Structure of POSSDAU-MR

Table S1. NMR results for POSSDAU-MR.

Type of Substitution	Signal	The Value of Chemical Shift [ppm] ^{1}H-NMR [DMSO-d6, 295K, 500MHz]	The Value of Chemical Shift [ppm] ^{13}C-NMR [DMSO-d6, 295K, 500MHz]
Unsubstituted POSS Corner	1(P)	1.02	13.1
	2(P)	2.63	25.7
	3(P)	2.56	33.9
	4(P)	3.53	61.4
**Substituted POSS Corner**	1(P)	1.02	13.1
	2(P)	2.63	25.7
	3(P)	2.75	29.8
	4(P)	4.14	63.4
**Substituted and Unsubstituted PEG**	1(PEG)	-	-
	2(PEG)	2.45/2.56	35.3/34.0
	3(PEG)	3.73/3.60	65.7/66.9
	4(PEG)	3.51/3.52	70.3/70.1
	5(PEG)		
	6(PEG)		

	7(PEG)	3.43	71.8
	8(PEG)	3.25	58.6

Table S2. Concentrations of the reagents used in conjugation reaction **4–9**.

Products	Reactants	m [g]	n [mol]	V [ml]
***PossDoxPEG1* (4)**	SAMDOX	0.1366	$2.1193 \cdot 10^{-4}$	-
	NHS_{SAMDOX}	0.0366	$3.1801 \cdot 10^{-4}$	-
	EDC_{SAMDOX}	0.0610	$3.1821 \cdot 10^{-4}$	-
	DMF_{SAMDOX}	-	-	9.0
	PEG1	0.1000	$1.6987 \cdot 10^{-4}$	-
	NHS_{PEG1}	0.0293	$2.5458 \cdot 10^{-4}$	-
	EDC_{PEG1}	0.0488	$2.5456 \cdot 10^{-4}$	-
	DMF_{PEG1}	-	-	9.0
	POSS	0.0438	$2.1193 \cdot 10^{-5}$	-
	DMF_{POSS}	-	-	9.0
***PossDoxPEG2* (5)**	SAMDOX	0.1366	$2.1224 \cdot 10^{-4}$	-
	NHS_{SAMDOX}	0.0366	$3.1801 \cdot 10^{-4}$	-
	EDC_{SAMDOX}	0.0610	$3.1821 \cdot 10^{-4}$	-
	DMF_{SAMDOX}	-	-	9.0
	PEG2	0.2229	$1.0614 \cdot 10^{-4}$	-
	NHS_{PEG2}	0.0183	$1.5900 \cdot 10^{-4}$	-
	EDC_{PEG2}	0.0305	$1.5910 \cdot 10^{-4}$	-
	DMF_{PEG2}	-	-	9.0
	POSS	0.0365	$1.7660 \cdot 10^{-5}$	-
	DMF_{POSS}	-	-	9.0
***PossDoxPEGB3* (6)**	SAMDOX	0.0343	$5.3294 \cdot 10^{-5}$	-
	NHS_{SAMDOX}	0.0092	$7.9937 \cdot 10^{-5}$	-
	EDC_{SAMDOX}	0.0153	$7.9812 \cdot 10^{-5}$	-
	DMF_{SAMDOX}	-	-	9.0
	PEGB3	0.0800	$2.6667 \cdot 10^{-5}$	-
	NHS_{PEGB3}	0.0046	$3.9969 \cdot 10^{-5}$	-
	EDC_{PEGB3}	0.0077	$4.0167 \cdot 10^{-5}$	-
	DMF_{PEGB3}	-	-	9.0
	POSS	0.0095	$4.5966 \cdot 10^{-6}$	-
	DMF_{POSS}	-	-	9.0
***PossDauPEG1* (7)**	SAMDAU	0.1333	$2.1239 \cdot 10^{-4}$	-
	NHS_{SAMDAU}	0.0367	$3.1888 \cdot 10^{-4}$	-
	EDC_{SAMDAU}	0.0612	$3.1925 \cdot 10^{-4}$	-
	DMF_{SAMDAU}	-	-	9.0
	PEG1	0.0675	$1.1466 \cdot 10^{-4}$	-

	NHS_{PEG1}	0.0293	$1.5284 \cdot 10^{-4}$	-
	EDC_{PEG1}	0.0488	$2.5456 \cdot 10^{-4}$	-
	DMF_{PEG1}	-	-	9.0
	POSS	0.0438	$2.1193 \cdot 10^{-5}$	-
	DMF_{POSS}	-	-	9.0
***PossDauPEG2* (8)**	SAMDAU	0.1332	$2.1224 \cdot 10^{-4}$	-
	NHS_{SAMDAU}	0.0367	$3.1888 \cdot 10^{-4}$	-
	EDC_{SAMDAU}	0.0611	$3.1873 \cdot 10^{-4}$	-
	DMF_{SAMDAU}	-	-	9.0
	PEG2	0.2229	$1.0614 \cdot 10^{-4}$	-
	NHS_{PEG2}	0.0183	$1.5901 \cdot 10^{-4}$	-
	EDC_{PEG2}	0.0305	$1.5910 \cdot 10^{-4}$	-
	DMF_{PEG2}	-	-	9.0
	POSS	0.0365	$1.7660 \cdot 10^{-5}$	-
	DMF_{POSS}	-	-	9.0
***PossDauPEGB3* (9)**	SAMDAU	0.0334	$5.3219 \cdot 10^{-5}$	-
	NHS_{SAMDAU}	0.0092	$7.9937 \cdot 10^{-5}$	-
	EDC_{SAMDAU}	0.0153	$7.9812 \cdot 10^{-5}$	-
	DMF_{SAMDAU}	-	-	2.5
	PEGB3	0.0800	$2.6667 \cdot 10^{-5}$	-
	NHS_{PEGB3}	0.0046	$3.9969 \cdot 10^{-5}$	-
	EDC_{PEGB3}	0.0077	$4.0167 \cdot 10^{-5}$	-
	DMF_{PEGB3}	-	-	2.5
	POSS	0.0095	$4.5966 \cdot 10^{-6}$	-
	DMF_{POSS}	-	-	2.5

Table S3. Concentrations of the conjugates **4–9** in drugs release study.

**Type of Nanoconjugate**	$M_{CONJUGATES}$	V_{BUFFER}	V_{DMF}	$C_{CONJUGATES}$
PossDoxPEG1 (4)	6.3 mg	80 mL	1 mL	0.07778 mg/mL
PossDoxPEG2 (**5**)	4.6 mg	50 mL	1 mL	0.09019 mg/mL
PossDoxPEGB3 (**6**)	5.2 mg	65 mL	1 mL	0.07879 mg/mL
PossDauPEG1 (**7**)	4.9 mg	50 mL	1 mL	0.09608 mg/mL
PossDauPEG2 (**8**)	6.7 mg	80 mL	1 mL	0.08272 mg/mL
PossDauPEGB3 (**9**)	1.4 mg	50 mL	1 mL	0.02745 mg/mL

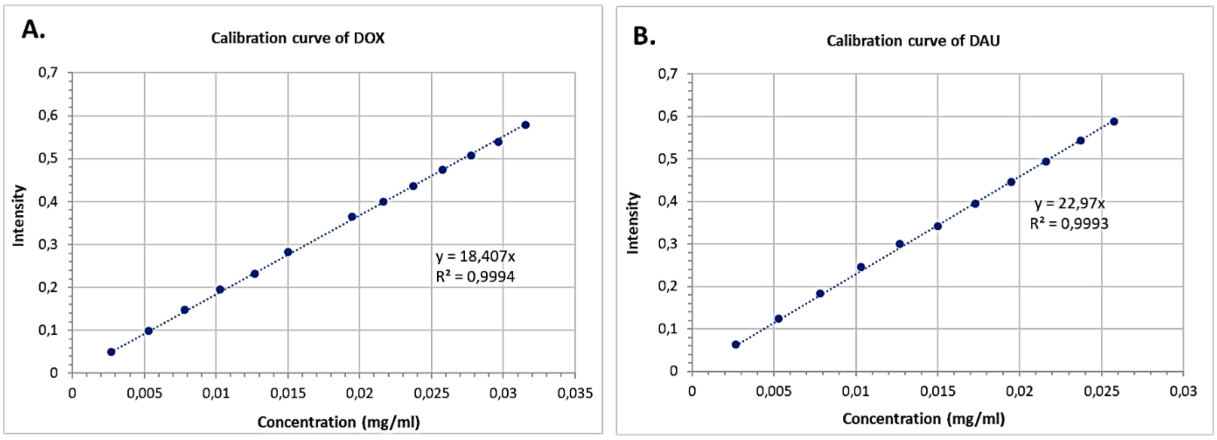

Fig. S4. Calibration curves (UV-VIS) for: (**A**) DOX in H_2O/DMF (5:1) (**B**) DAU in H_2O/DMF.

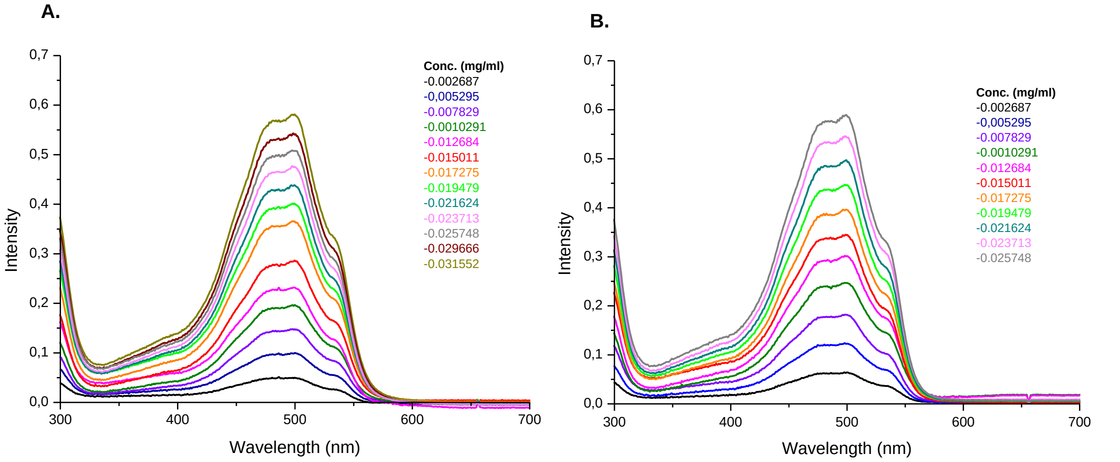

Fig. S5. Dependence of absorbance intensity on anthracycline concentration (**A**. DOX, **B**. DAU) (UV-Vis spectrum).

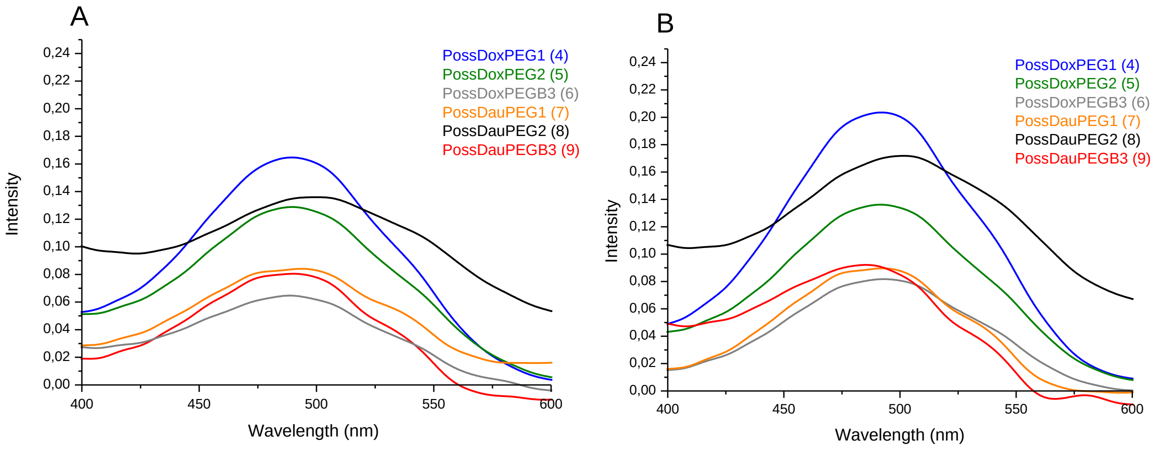

Fig. S6. A Study of DOX/DAU release from nanoconjugates at pH 5.5 at 310 K quantified by UV-Vis method after 21 h (**A**) and after 42 h (**B**).

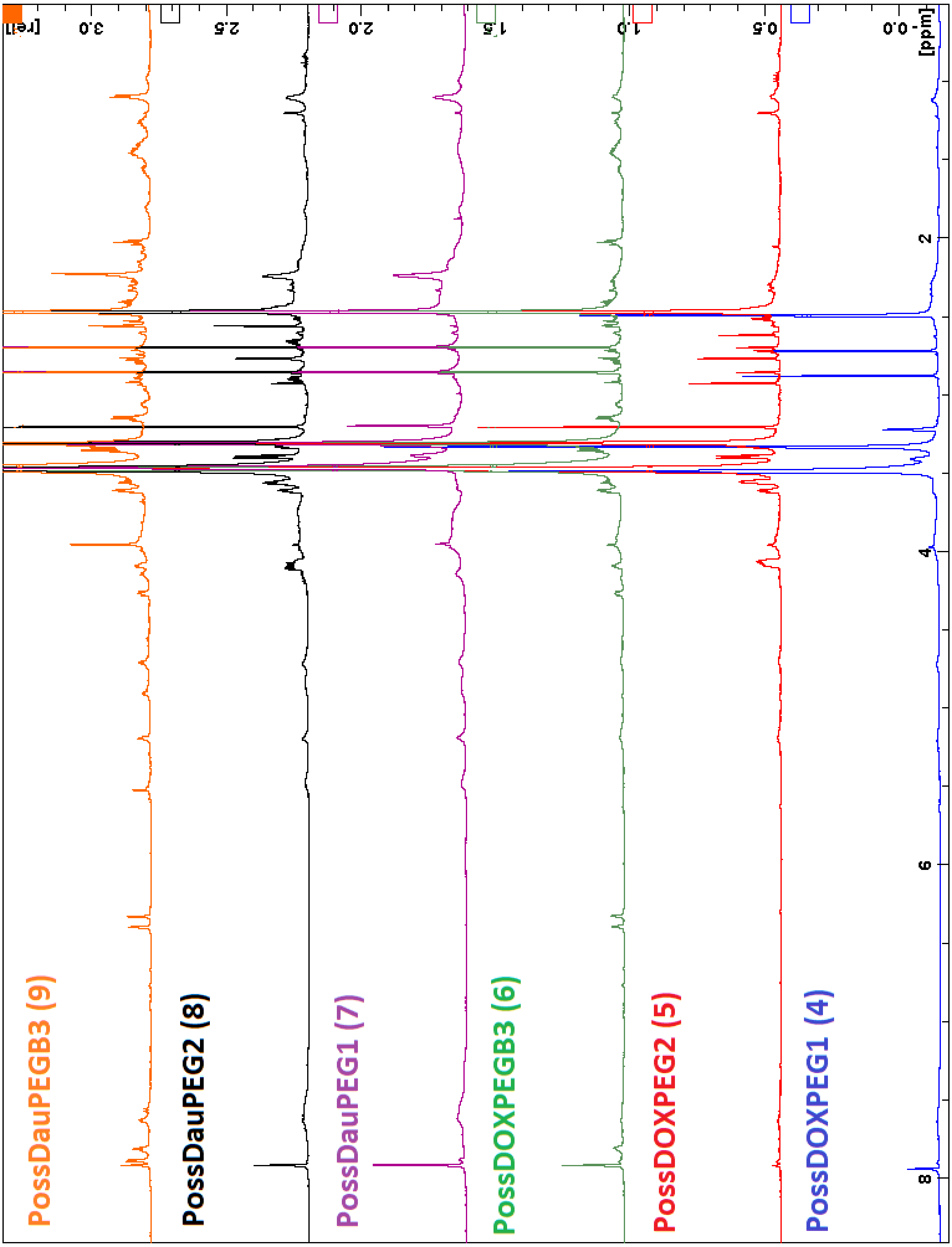

Fig. S7. ^{1}H-NMR spectra of **4–9** (500 MHz, 295 K, DMSO-d6).
